# Effect of metformin therapy on 2-h post-glucose insulin levels in patients of polycystic ovarian syndrome

**DOI:** 10.4103/0974-1208.74156

**Published:** 2010

**Authors:** Pikee Saxena, Anupam Prakash, Aruna Nigam

**Affiliations:** Department of Obstetrics and Gynecology, Lady Hardinge Medical College and Smt. Sucheta Kriplani Hospital, New Delhi, India; 1 Department of Medicine, Lady Hardinge Medical College and Smt. Sucheta Kriplani Hospital, New Delhi, India

**Keywords:** Metformin, hyperinsulinemia, PCOS, 2-h post glucose insulin

## Abstract

**AIMS:**

To evaluate if 2-h post glucose insulin level is an effective tool to monitor insulin resistance in response to metformin therapy, in infertile women with polycystic ovarian syndrome (PCOS).

**SETTINGS AND DESIGN:**

This prospective observational study was carried out in a tertiary care infertility clinic.

**MATERIALS AND METHODS:**

40 women with PCOS were categorized as having insulin resistance if fasting or 2-h post glucose insulin levels were >25 or >41μU/ml respectively. Post glucose insulin was compared before and after six months of metformin therapy along with other clinical, hormonal and metabolic parameters by using McNemar and the Student’s t-test.

**RESULTS:**

Fasting insulin was elevated in 4 (10%) and post-load insulin in 34 (85%) patients; after metformin therapy respective values were 2 (5%) and 16 (40%). Metformin therapy reduced post glucose insulin levels (*P*<0.001), improved the regularity of periods (*P*<0.001) and resulted in reduction of LH levels (*P*<0.001), total testosterone (*P*<0.001) and mean Body mass index (BMI) (*P*=0.047). Metformin therapy did not alter waist-hip ratio and fasting insulin levels.

**CONCLUSION:**

2-h post glucose insulin level is an effective tool to monitor insulin resistance in PCOS patients and improves significantly after metformin therapy, similar to improvements observed in clinical, hormonal and metabolic parameters.

## INTRODUCTION

Polycystic ovarian syndrome (PCOS) is one of the most common endocrinopathy in women of reproductive age group. Most women with PCOS suffer from insulin resistance and the resulting hyperinsulinemia plays a key role in the pathogenesis of reproductive disorders.[1] It has been seen that hyperinsulinemia associated with insulin resistance has been linked to all features of the syndrome like hyperandrogenism, irregular cycles, infertility, acne, hirsutism and metabolic disturbances.[2] It has been demonstrated that by reducing hyperinsulinemia, insulin-lowering agents might improve endocrine, metabolic and reproductive abnormalities in PCOS patients and have numerous beneficial effects.[2] Metformin is currently the preferred insulin-sensitizing drug for treatment of PCOS and has been shown to improve the clinical, hormonal and metabolic profile of women with PCOS.[3]

Tests to identify insulin resistance are difficult and inconvenient to perform in clinical practice. A simple and reliable test will be useful to screen and then to monitor these patients so that dose of insulin sensitizers can be adjusted accordingly. 2-h post-load glucose[4] is one such measurement which in recent times is gaining importance as a one-step, single and reliable test for determining insulin resistance. The present study has been planned to evaluate 2-h post-glucose insulin levels at the time of diagnosis of PCOS and after six months of therapy with metformin and to determine its relation with other clinical, biochemical and metabolic markers dependent on insulin resistance in infertile PCOS patients.

## MATERIALS AND METHODS

Selection and description of participants: This is an observational clinical study which was performed in an infertility clinic of a tertiary care center. 40 consecutive patients with PCOS were recruited after a written informed consent. Patients were labeled as having PCOS according to the Rotterdam criteria.[5] Women suffering from diabetes mellitus, on steroid hormones, on drugs known to have effects on lipid metabolism during the past 2 years, age < 18 years or > 38 years were not included in the study.

Technical information: A detailed history was taken and complete physical examination performed. Ferriman Gallawey scoring of ≥ 7 was used for grading hirsutism.[6] Anthropometric measurements were made by the same observer to obviate inter-observer variation. Pelvic ultrasound both transabdominal and transvaginal were performed to record the status of ovaries, uterus and adenexa. Ovaries were carefully evaluated for multiple (>10) immature follicles of 2 to 8 mm diameter and stromal hyperplasia with/without hyperemia. Endometrial thickness was also measured in the early follicular phase.

Sample collection and estimation: Patients were advised to have carbohydrate diet of at least 300 g for three days prior to the test. The fasting blood sample was analyzed for serum lipid profile, follicle stimulating hormone (FSH), luteinizing hormone (LH), thyroid stimulating hormone (TSH), prolactin and testosterone on day 2 or 3 of menstrual cycle while serum progesterone was measured on day 21. Blood sugar values and serum insulin values were determined during a 75 g oral glucose tolerance test (GTT). Values of GTT were assessed according to ADA, 1997.[7] Presence of insulin resistance was defined by fasting or post-load insulin levels > 25 and > 41μU/ml respectively (DRG diagnostic instrument, GmbH, Germany). Hormonal estimation was done by chemiluminescence assay. Lipid profile was estimated by using enzymatic colorimetric technique and criteria adopted were in consonance with NCEP-ATP III guidelines.[8]

All PCOS women were treated with metformin 500 mg eight hourly for three months after baseline investigations. After three months, fasting and 2-h post-glucose load serum insulin levels were estimated for each patient and the dose of metformin was titrated accordingly to 850 or 1000 mg 12 hourly if insulin levels remained high. Metformin was further continued and after the designated treatment period of six months repeat clinical, hormonal and metabolic assessment was done along with post glucose insulin test and was compared with pre-treatment values.

### Statistical analysis

Statistical analysis was performed on the Statistical Package for the Social Sciences (SPSS version 10) software (SPSS, Chicago, IL, USA). Insulin levels, clinical, hormonal and metabolic parameters were compared before and after therapy in the PCOS women employing the McNemar test and the two-tailed paired Student’s *t*-test. *P* value < 0.05 was taken as the cut-off level for significance.

## RESULTS

Mean age of the patients was 29.4 ± 2.2 years. 8 (20%) patients had a family history of diabetes, 4 (10%) had impaired GTT, 12 (30%) had deranged lipid profile and 8 (20%) had hypertension. Fasting insulin > 25 μU/ml was detected in 4 (10%) and post-load insulin level of >41 μU/ml was observed in 34 (85%) patients i.e. 85% had insulin resistance. After metformin therapy, high fasting insulin levels were notable in 2 (5%) and high post-load insulin levels in only 16 (40%) subjects. As regards the diagnostic criteria for PCOS, menstrual irregularity was noted in 32 cases (80%); clinical hyperandrogenism was found in 31 (77.5%) and biochemical hyperandrogenism (serum testosterone value >1 ng / dl) was present in 32 (80%) subjects; pelvic ultrasound showed polycystic appearance of ovaries in all subjects (100%). After six months of therapy, 52% of the women previously having irregular cycles attained normal menstrual pattern, hirsutism score reduced significantly from 13.8± 3.3 to 9.2 ± 2.2. Ultrasonic picture of polycystic ovaries, endometrial hyperplasia reduced significantly among clinical parameters [Table 1]. Significant reductions in serum LH and testosterone values were observed while serum progesterone levels increased after metformin therapy. Serum FSH, prolactin and TSH levels were unaltered [Table 2].

**Table 1:** Clinical features of study group before and after metformin therapy

Indicators	Pre treatment *n*=40	Post treatment *n*=40	Significance *P* value
Mean hirsuitism Score (mean ± SD)	13.8 ± 4.2	9.2 ± 2.2	< 0.001
Irregular cycles	32 (80)	11 (28)	<0.001
Acne	8 (20)	4 (10)	0.133
Abnormal pelvic ultrasound	40 (100)	8 (20)	<0.001
Endometrial thickness > 4mm	6 (15)	Nil	0.041
Acanthosis nigricans	5 (12.5)	5 (12.5)	1.0

NS = Not significant, Figures in parentheses are in percentage

**Table 2:** Hormonal profile of the study group before and after therapy

Hormone tested	Pre treatment *n*=40	Post treatment *n*=40	Significance *P* value
LH (mIU/ml)	11.9±3.4	7.2±2.6	< 0.001
Testosterone (ng/dl)	1.4±0.6	0.7 ±0.4	< 0.001
Progesterone (ng/dl)	2.3 ±1.4	5.9 ±1.4	< 0.001
FSH (mIU/ml)	5.7± 2.6	4.5±2.7	0.053
Prolactin (ng/dl)	16.5±6.0	14.3±4.7	0.055
TSH (mU/ml)	3.4±2.5	3.9±1.7	0.279

NS = Not significant

Metabolic parameters before and after metformin therapy are depicted in Table 3. After metformin therapy, BMI, post-glucose insulin values and serum triglyceride levels reduced significantly. Significant change was not observed in fasting or post-load glucose, fasting insulin values and other parameters of lipid profile.

**Table 3:** Metabolic parameters in the study group before and after therapy

Indicators	Pre treatment *n*=40	Post treatment *n*=40	Significance *P* value
Body mass index	25.5±4.4	23.6±3.7	0.047
Fasting glucose (mg/dl)	88.3±14.8	83.72± 9.8	0.059
2-h post load glucose (mg/dl)	92.9±17.1	85.2±16.3	0.060
Waist/hip ratio	0.89±0.07	0.85±0.07	0.055
Fasting serum insulin (μU/ml)	14.3±6.4	11.8±5.0	0.060
Post-glucose serum insulin (μU/ml)	76.1±34.3	42.6±20.9	<0.001
Serum triglycerides (mg/dl)	138.4±47.5	104.9±35.1	<0.001
Serum cholesterol (mg/dl)	177.7±39.7	173.3±37.6	0.621
Serum HDL (mg/dl)	39.3±13.1	42.6±18.3	0.449
Serum LDL (mg/dl)	124.8±45.2	115.9±36.5	0.214

Values given are mean ± SD, NS = Not significant

## DISCUSSION

Insulin resistance is defined as decreased ability of insulin to stimulate glucose disposal into the target tissue or a reduced glucose response to a given amount of insulin. Since cells need glucose for their survival, more insulin is produced by the pancreas resulting in hyperinsulinemia. In clinical practice, no single laboratory test is used to diagnose insulin resistance. Diagnosis is based on clinical findings corroborated with laboratory tests. Individual patients are screened based on the presence of co-morbid conditions.

For detecting insulin resistance, numerous techniques such as euglycemic clamp technique, insulin tolerance test, insulin sensitizing test, IV/oral glucose tolerance test, fasting insulin levels, fasting glucose to insulin ratio have been devised by several investigators. However, all these techniques are time consuming, stressful to the patients or are applicable to selective group of subjects and are not suitable for wide clinical use.

It was observed that insulin levels were much higher 2 h after 75 g glucose load in PCOS subjects[4] compared to fasting insulin levels. It is apparent that for maintaining glucose values within normal range, a much higher level of insulin is needed in PCOS subjects due to insulin resistance and therefore, hyperinsulinemia is a common association with PCOS. A significant reduction in post-glucose insulin values was seen after treatment with metformin, an insulin sensitizer, which is known to reduce insulin resistance and accordingly improves clinical, hormonal and metabolic parameters.[3] Metformin therapy showed significant improvement in BMI but WHR was not improved in this study as also seen in the study of Velazquez *et al*.,[9] Most important change was seen in the menstrual pattern and 52% of women having irregular menstrual cycle achieved regular cycles. Hirsutism and acne improved which is comparable to study of Ibanez *et al*.,[10] Previous coarse hairs were replaced by fine, softer hairs with slower growth after metformin therapy. Hormonal assessment showed reduction in serum LH and serum testosterone level and increase in day 21 serum progesterone levels. These results are comparable to the study of Velazque *et al*.,[9] Present study revealed that metformin administration resulted in significant decrease in fasting triglyceride levels while study of Glueck *et al*.,[11] shows long term treatment with metformin yields a moderate improvement in plasma triglyceride and LDL cholesterol concentrations.

## CONCLUSION

To conclude, a 2-h post glucose insulin level is useful to detect and monitor all PCOS patients with insulin resistance. 2-h post glucose insulin levels show significant improvement in response to metformin therapy and parallel the improvements seen in the clinical, hormonal and metabolic parameters of these patients.

## References

[1] Franks S (1995). Polycystic ovary syndrome. N Engl J Med.

[2] De Leo V, Musacchio MC, Palermo V, Di Sabatino A, Morgante G, Petraglia F (2009). Polycystic ovary syndrome and metabolic co-morbidities: therapeutic options. Drugs Today.

[3] Otta CF, Wior M, Iraci GS, Kaplan R, Torres D, Gaido MI (2010). Clinical, metabolic, and endocrine parameters in response to metformin and lifestyle intervention in women with polycystic ovary syndrome: A randomized, double-blind, and placebo control trial. Gynecol Endocrinol.

[4] Mathur R, Alexander CJ, Yano J, Trivax B, Azziz R (2008). Use of metformin in polycystic ovary syndrome. Am J Obstet Gynecol.

[5] (2004). Rotterdam ESHRE/ASRM-Sponsored PCOS consensus workshop group. Revised 2003 consensus on diagnostic criteria and long-term health risks related to polycystic ovary syndrome (PCOS). Hum Reprod.

[6] Falsetti L, Gambera A, Andrico S, Sartori E (2002). Acne and hirsutism in polycystic ovary syndrome: clinical, endocrine-metabolic and ultrasonographic differences. Gynecol Endocrinol.

[7] (1997). Report of the Expert Committee on the diagnosis and classification of diabetes mellitus. Diabetes Care.

[8] Tong PC, Kong AP, So WY, Yang X, Ho CS, Ma RC (2007). The usefulness of the International Diabetes Federation and the National Cholesterol Education Program’s Adult Treatment Panel III definitions of the metabolic syndrome in predicting coronary heart disease in subjects with type 2 diabetes. Diabetes Care.

[9] Velazquez EM, Mendoza S, Hamer T, Sosa F, Glueck CJ (1994). Metformin therapy in polycystic ovary syndrome reduces hyperinsulinemia, insulin resistance, hyperandrogenemia, and systolic blood pressure, while facilitating normal menses and pregnancy. Metabolism.

[10] Ibanez L, Valls C, Potau N, Marcos MV, de Zegher F (2000). Sensitization to insulin in adolescent girls to normalize hirsutism, hyperandrogenism, oligomenorrhea, dyslipidemia, and hyperinsulinism after precocious pubarche. J Clin Endocrinol Metab.

[11] Glueck CJ, Wang P, Fontaine R, Tracy T, Sieve-Smith L (1999). Metformin-induced resumption of normal menses in 39 of 43 (91%) previously amenorrheic women with the polycystic ovary syndrome. Metabolism.

